# Walking to work in Canada: health benefits, socio-economic characteristics and urban-regional variations

**DOI:** 10.1186/1471-2458-11-212

**Published:** 2011-04-04

**Authors:** Peter Kitchen, Allison Williams, James Chowhan

**Affiliations:** 1 School of Geography & Earth Sciences, McMaster University, Hamilton, Ontario, Canada; 2 DeGroote School of Business, McMaster University, Hamilton, Ontario, Canada

## Abstract

**Background:**

There is mounting concern over increasing rates of physical inactivity and overweight/obesity among children and adult in Canada. There is a clear link between the amount of walking a person does and his or her health. The purpose of this paper is to assess the health factors, socio-economic characteristics and urban-regional variations of walking to work among adults in Canada.

**Methods:**

Data is drawn from two cycles of the Canadian Community Health Survey: 2001 and 2005. The study population is divided into three groups: non-walkers, lower-duration walkers and high-duration walkers. Logistic regression modeling tests the association between levels of walking and health related outcomes (diabetes, high blood pressure, stress, BMI, physical activity), socio-economic characteristics (sex, age, income, education) and place of residence (selected Census Metropolitan Areas).

**Results:**

In 2005, the presence of diabetes and high blood pressure was not associated with any form of walking. Adults within the normal weight range were more likely to be high-duration walkers. Females and younger people were more likely to be lower-duration walkers but less likely to be high-duration walkers. There was a strong association between SES (particularly relative disadvantage) and walking to work. In both 2001 and 2005, the conditions influencing walking to work were especially prevalent in Canada's largest city, Toronto, as well as in several small to medium sized urban areas including Halifax, Kingston, Hamilton, Regina, Calgary and Victoria.

**Conclusion:**

A number of strategies can be followed to increase levels of walking in Canada. It is clear that for many people walking to work is not possible. However, strategies can be developed to encourage adults to incorporate walking into their daily work and commuting routines. These include mass transit walking and workplace walking programs.

## Background

There is growing concern in Canada over increasing rates of overweight/obesity and declining levels of physical fitness among children and adults [1-4]. The negative health consequences of this situation and its economic burden on the health care system have been documented [5,6]. Federal and provincial governments have embarked on programs and strategies aimed at improving levels of physical fitness [7,8]. There is a clear link between the amount of walking a person does and his or her health [9-11]. Research has examined the effect of walking on controlling diabetes [12], preventing heart [13] and cardiovascular disease [14] and decreasing the risk of obesity [15]. In recent years, the concept of the *walkable city *has also garnered attention [16-19]. Walking is a popular and simple form of exercise performed on a daily basis by millions of Canadians, including those for whom it is a means of transportation. In the 2006 Census, about 900,000 people aged 15 and over, representing 7 percent of all commuters, reported that they walk to work. Table 1 shows the counts and percentage of individuals using walking as a mode of transportation in Canada and 27 Census Metropolitan Areas (CMAs) in 2001 and 2006. While walking has been recognized as important to maintaining and improving health, there is a need for more research into walking to work. Very few studies in Canada, or internationally, have investigated this mode of transportation, which has the potential to be promoted as an effective policy for addressing the problems of physical inactivity and overweight/obesity among adults.

**Table 1:** Walking as a Mode of Transportation: Individuals Employed in the Labour Force Aged 15 Years Old and Over, Having a Usual Place of Work, and Living in Canada and Selected Census Metropolitan Areas (CMAs), 2001 and 2006

	2001 Census		2006 Census	
	Walked	% of commuters	Walked	% of commuters
Canada	844,315	6.9	894,990	6.8
St. John's	4,500	5.9	5,200	6.6
Halifax	17,520	10.3	18,250	10.3
Saint John	3,670	6.9	3,860	7.5
Saguenay	--	--	3,305	5.3
Québec	22,760	7.0	25,955	7.6
Sherbrooke	5,090	7.2	6,270	7.8
Trois-Rivieres	3,485	6.0	3,665	6.1
Montréal	92,955	5.9	95,490	5.9
Ottawa-Gatineau	35,695	6.8	37,680	7.0
Kingston	6,790	10.4	7,070	10.4
Oshawa	5,150	3.6	4,975	4.6
Toronto	102,365	4.6	109,945	4.7
Hamilton	15,645	5.1	15,320	5.8
St. Catharines	8,370	5.0	8,445	5.5
Kitchener	10,135	4.9	11,680	5.4
London	11,810	5.9	12,720	6.2
Windsor	6,405	4.7	5,430	4.0
Sudbury	4,410	6.5	4,205	6.3
Thunder Bay	2,935	5.4	3,190	6.1
Winnipeg	20,015	6.1	19,135	5.9
Regina	4,900	5.2	5,625	5.9
Saskatoon	6,105	5.8	6,840	6.4
Calgary	29,260	5.9	30,240	5.8
Edmonton	22,025	4.7	26,280	5.5
Abbotsford	2,230	3.6	2,130	4.2
Vancouver	58,705	6.5	60,275	6.8
Victoria	14,565	10.4	15,765	11.1

Source: Statistics Canada 2001 and 2006 Census.

The purpose of this paper is to contribute to the research by exploring the relationship between health conditions, socio-economic characteristics and urban-regional variations of walking to work in Canada. A paper published in 2007 made an important contribution by examining the factors associated with active transportation in Canada, namely walking and cycling [20]. That research is used as a guide for this paper, which takes a different approach by investigating patterns and trends of walking to work among adults aged 20 to 64 in 27 urban areas in 2001 and 2005. Clearly, walking to work is only possible in situations where the built environment facilitates it (e.g. sufficient sidewalks and safe intersections), where the distance between a person's home and place of work is not too great, and where time permits. However, as will be outlined in the discussion section, several strategies can be pursued to encourage people who commute to work by other means (e.g. vehicle or public transit) to incorporate walking into their daily routines.

## Methods

The data for this research are drawn from the 2001 and 2005 Canadian Community Health Survey (CCHS) master file. The CCHS is an annual cross-sectional survey of Canadians aged 12 or over in all provinces and territories. Its primary objective is to gather health-related data on a wide range of topics and issues. The CCHS is made available to researchers in two forms: a Public Use Micro Data File (PUMF) and a master file. The master file contains micro data that is not available in the PUMF, including the CMA where each survey respondent resides. The data in the master file is not openly available to researchers, although it can be accessed following an application process. The authors were granted permission to use the CCHS master files through Statistics Canada's Research Data Centres Program, a process adjudicated by the Social Sciences and Humanities Research Council. A formal ethics application is not required.

The CCHS is a large survey where all data are self-reported and the sample is representative of the Canadian household population. The 2001 survey has a sample size of 130,880 while the 2005 survey has a sample size of 132,221. Using two cross-sections permits the verification of the robustness of the results enabling them to be generalized over time; alternatively, using only one cross-section would be accompanied by the limitation that any results could be spurious.

The CCHS includes a question on the number of hours per week a respondent spent walking to work, school or for errands. The variable is listed in the survey as follows: Number of hours walking - to work or to school. The question posed in the survey is as follows: In a typical week in the past 3 months, how many hours did you usually spend walking to work or to school or while doing errands? The responses to this question are as follows: None -- Less than 1 hour--From 1 to 5 hours--From 6 to 10 hours--From 11 to 20 hours--More than 20 hours. For the purpose of this research this variable was recoded into three categories: 0 hours (no walking), between 0 to 5 hours, and 6 hours or more. The decision to employ these walking cut-points was based on how the response categories are grouped in the CCHS (see question above) and the desire to assess progressive change across the non-walking to walking continuum. As explained below, in order to achieve some health benefits, it is recommended that a person walk at least 2.5 hours a week with this figure situated exactly in the middle of the second category (0 to 5 hours). The third category (6 hours or more) represents people who exceed the minimal requirement of 2.5 hours. Furthermore, a recent study of active transportation analyzed data from the CCHS and also employed a cut-point of 6 or more hours of walking per week [20].

As stated, the objective is to examine characteristics associated with walking to work. In an effort to capture the portion of study population which is employed and to exclude the portion which is attending school, the data analysis was carried out on a sample of respondents aged 20 to 64 who worked in a job or business in the week prior to the survey being conducted. While high school students are not included in the sample, it is likely that some university or college students who are combining work and study are included.

Two key variables were used to implement the above selection criteria for the study: 1) Age (20 to 64), and 2) Work Status (i.e. Worked at a job or business last week) (Yes). The resulting sample sizes are 51,540 in 2001 (39.0% of all CCHS respondents) and 50,082 in 2005 (37.9% of all CCHS respondents). The CCHS variables used in the data analysis (and their categories) are listed in Table 2 and comprise three types: 1) health-related (diabetes, high-blood pressure, stress, BMI, physical activity^1^); 2) socio-economic (sex, age, income, education); and 3) urban-regional (CMAs). The selection of these variables is based on a review of the literature. Other potential measures influencing the decision to walk to work, such as: type of profession, number of cars in a household, access to public transit, and having a driver's license, were not included in the analysis as these questions were not asked in the CCHS.

**Table 2:** Characteristics of the Study Samples (Adults 20 to 64 and Currently Working)*

	2001			2005		
Variable	Total sample	Non-walkers	All walkers	Total sample	Non-walkers	All walkers
Walking (hrs per week)						
0 hrs (no walking)	37.0	100	--	30.0	100	--
0-5 hrs	40.0	--	63.6	45.5	--	64.9
6 hrs or more	23.0	--	36.4	24.5	--	35.1
Has diabetes						
Yes	2.2	2.1	2.2	2.8	3.1	2.7
No	97.8	97.9	97.8	97.2	96.9	97.3
Has high blood pressure						
Yes	8.0	8.3	7.8	10.1	10.9	9.7
No	92.0	91.7	92.2	89.9	89.1	90.3
Self-perceived stress						
Not all/not very	26.5	26.7	26.3	26.7	27.4	26.4
A bit	43.4	41.5	44.6	45.3	43.0	46.3
Quite a bit/extremely	30.1	31.8	29.1	28.0	29.6	27.3
Adult weight (BMI)						
Normal weight	51.2	49.6	52.1	48.5	46.4	49.4
Overweight/obese	48.8	50.4	47.9	51.5	53.6	50.6
Physical activity index						
Active/moderate	55.5	58.2	53.9	49.1	52.6	47.7
Inactive	44.5	41.8	46.1	50.9	47.4	52.3
Sex						
Male	54.6	59.5	51.7	55.3	60.5	53.1
Female	45.4	40.5	48.3	44.7	39.5	46.9
Age						
20-34	33.6	30.7	35.3	31.3	28.4	32.6
35-49	45.6	47.1	44.7	43.9	45.2	43.3
50-64	20.8	22.2	20.0	24.8	26.4	24.1
Household Income						
Under $20,000	6.3	5.3	6.9	4.0	3.2	4.4
$20,000 to $49,999	29.3	28.7	29.8	23.0	22.3	23.3
$50,000 to $79,999	32.2	32.5	31.9	29.7	30.3	29.5
$80,000 and over	32.2	33.5	31.4	43.3	44.2	42.8
Education						
Less than high school	5.6	6.5	5.1	3.2	3.9	2.9
High school	12.8	12.5	12.9	9.3	9.5	9.2
College or trades diploma	45.2	45.7	45.0	45.2	46.9	44.5
University	36.4	35.3	37.0	42.3	39.7	43.4

* All estimates are reported as percentages and for each variable the frequencies sum to 100%.

The data analysis involves logistic regression modeling to identify factors associated with walking to work. For each study year (2001 and 2005) three models are developed in which the progressive duration of walking to work is measured according to three categories: no walking, lower-duration walking and high-duration walking. In the first model, the dependent variable is dichotomized into all walkers (coded as 1) and non-walkers (coded as 0). In the second model, the dependent variable is dichotomized into lower-duration walkers (0 to 5 hours per week, coded as 1) and non-walkers (coded as 0). In the third model, the dependent variable consists exclusively of walkers and is dichotomized into high-duration walkers (6 or more hours per week, coded as 1) and lower-duration walkers (0 to 5 hours per week, coded as 0).

Three separate logistic regressions were run so that the differential relationships between the independent variables and the dependent variable at different points in the walking to work distribution could be better understood. In particular, Model 1 enables an understanding of the factors associated with the decision to walk or not to walk. Further, Models 2 and 3 enable an understanding of how the independent variables vary with lower and high duration walking. This approach was preferred over an ordered logistic regression because the ordered logistic model does not provide estimates at different points of walking duration but rather makes an assumption of parallel slopes across the cut-points of comparison. Similarly, the ordered logistic model by itself would not enable an understanding of which factors effect the decision to walk or not to walk.

In logistic regression, odds ratios compare the probability of events for two groups, where an odds ratio of 1 implies an event that is equally likely to occur in one group as it is in the other group. An odds ratio greater than 1 implies the event is more likely to occur in the comparison group than the reference group. Further, an odds ratio less than 1 means the event is less likely in the comparison group than the reference group. For all analysis, the CCHS individual sample weight is used to adjust for bias due to the complex survey design and unequal probability of selection. Bootstrap techniques are employed to ensure appropriate inference by correcting for downward bias standard errors and adjusting for intra-cluster correlation, again due to the survey's complex design [21]. Using the sample survey weights implies that all estimates can be considered representative of the Canadian survey population. All analysis is conducted using the statistical software Stata 11 (http://www.stata.com).

## Results

Table 3 displays the results of the three logistic regression models for 2001. Independent variables having significant odds ratios (*-p < 0.10, **-p < 0.05 and ***-p < 0.01) are marked with asterisks. In Model 1 (walking/no walking), three health-related variables were strongly associated with walking to work. Interestingly, people with diabetes were 1.2 times more likely to walk to work than those without diabetes (OR = 1.22; 95% CI: 1.01, 1.45). Those with 'quite a bit/extremely' stressful lives were less likely to walk than people having 'not at all/not very' stressful lives (OR = 0.92, 95% CI: 0.86, 1:00). Not surprisingly, physically active people were 1.2 times more likely to walk to work than those who are physically inactive (OR = 1.20, 95% CI: 1.13, 1.27). In terms of socio-economic status and confirming the results of earlier research, females were more likely to walk to work than males (OR = 1.38, 95% CI = 1.13, 1.46) and people aged 20 to 34 were more likely to walk than those aged 50 to 64 (OR = 1.20, 95% CI = 1.11, 1.29). In addition, there was a strong association between household income and walking to work. Those with an income below $50,000 were more likely to walk than people with a household income of $80,000 or over (under $20,000, OR = 1.56, 95% CI: 1.38, 1.76; $20,000 to $49,999, OR = 1.23, 95% CI = 1.14, 1.33). People without a high school education were less likely to walk than those with a university degree (OR = 0.74, 95% CI: 0.65, 0.83). With respect to urban-regional variations, Model 1 indicates that the conditions influencing walking to work in 2001 were especially prevalent in Canada's largest CMA, Toronto (OR = 1.46, 95% CI: 1.30, 1.64), as well as in several small to medium-sized urban areas, including Halifax (OR = 1.82, 95% CI: 1.39, 2.38), Hamilton (OR = 1.75, 95% CI: 1.33,2.29), Regina (OR = 2.73, 95% CI: 1.95,3.81), Calgary (OR = 3.31, 95% CI: 2.69, 4.06) and Victoria (OR = 3.17, 95% CI: 2.06, 4.86). These cities were more likely to have people walking to work than the reference group - people who do not reside in a CMA.

**Table 3:** Results of Logistic Regression Analyses: Walking to Work, Adults aged 20 to 64 and Currently Working, 2001 ^a^

	Model 1 Walking/No Walking (all walkers = 1 non-walkers = 0)		Model 2 Lower-Duration Walking/No Walking (0 to 5 hrs/wk= 1 non-walkers= 0)		Model 3 High-Duration Walking/Lower-Duration Walking (6 or more hrs/wk = 1 0 to 5 hrs/wk = 0)	
Independent Variables	Odds ratios	95% CI	Odds ratios	95% CI	Odds ratios	95% CI
**Has diabetes**						
Yes	1.22**	1.01, 1.45	1.30**	1.06, 1.59	0.79**	0.62, 0.99
No	Reference		Reference		Reference	
**Has high blood pressure**						
Yes	1.00	0.90, 1.11	1.03	0.92, 1.15	0.93	0.82, 1.05
No	Reference		Reference		Reference	
**Self-perceived stress**						
Not at all/not very	Reference		Reference		Reference	
A bit	1.06*	0.99, 1.13	1.07	0.99, 1.14	0.96	0.88, 1.04
Quite a bit/extremely	0.92**	0.86, 1.00	0.89**	0.82, 0.97	1.08	0.99, 1.18
**Adult BMI**						
Normal weight	1.00	0.94, 1.05	0.98	0.92, 1.04	1.07**	1.00, 1.15
Overweight/obese	Reference		Reference		Reference	
**Physical activity index**						
Active/moderate	1.20***	1.13, 1.27	1.16***	1.09, 1.23	1.09**	1.01, 1.16
Inactive	Reference		Reference		Reference	
**Sex**						
Male	Reference		Reference		Reference	
Female	1.38***	1.30, 1.46	1.45***	1.36, 1.54	0.89***	0.82, 0.95
**Age**						
20-34	1.20***	1.11, 1.29	1.25***	1.15,1.35	0.90**	0.82, 0.99
35-49	1.02	0.95, 1.09	1.04	0.96,1.12	0.94	0.86, 1.02
50-64	Reference		Reference		Reference	
**Household income**						
Under $20,000	1.56***	1.38, 1.76	1.39***	1.20,1.59	1.44***	1.24, 1.65
$20,000 to $49,999	1.23***	1.14, 1.33	1.17***	1.07,1.27	1.23***	1.11, 1.35
$50,000 to $79,999	1.12***	1.03, 1.20	1.09**	1.00,1.19	1.10**	1.00, 1.20
$80,000 and over	Reference		Reference		Reference	
**Education**						
Less than high school	0.74***	0.65, 0.83	0.69***	0.60,0.79	1.20**	1.03, 1.40
High School	0.96	0.88, 1.05	0.86**	0.77,0.95	1.40***	1.25, 1.55
College or trades	0.94*	0.87, 1.00	0.84***	0.78,0.90	1.38***	1.27, 1.49
University	Reference		Reference		Reference	
**Census Metropolitan Area**						
No CMA assigned	Reference		Reference		Reference	
St. John's	1.11	0.78, 1.56	1.46**	1.03, 2.06	0.40***	0.27, 0.58
Halifax	1.82***	1.39, 2.38	1.85***	1.38, 2.45	1.02	0.79, 1.30
Québec	0.44***	0.35, 0.55	0.53***	0.40, 0.67	0.64**	0.46, 0.87
Montréal	1.09	0.98, 1.21	1.48***	1.32, 1.66	0.36***	0.30, 0.42
Ottawa-Gatineau	0.86*	0.72, 1.02	1.07	0.89, 1.28	0.53***	0.42, 0.64
Toronto	1.46***	1.30, 1.64	1.56***	1.37, 1.76	0.89**	0.78, 1.00
Hamilton	1.75***	1.33, 2.29	1.79***	1.38, 2.30	0.98	0.78, 1.22
London	1.27**	1.04, 1.54	1.34**	1.09, 1.64	0.91	0.72, 1.14
Thunder Bay	0.46***	0.35, 0.61	0.62**	0.46, 0.83	0.39***	0.23, 0.66
Winnipeg	0.84*	0.69, 1.01	0.74**	0.60, 0.90	1.35**	1.07, 1.69
Regina	2.73***	1.95, 3.81	2.42***	1.67, 3.49	1.33**	0.99, 1.78
Saskatoon	1.15	0.88, 1.50	1.05	0.77, 1.42	1.22	0.89, 1.66
Calgary	3.31***	2.69, 4.06	2.29***	1.87, 2.81	2.09***	1.68, 2.60
Edmonton	1.01	0.83, 1.23	1.09	0.89, 1.32	0.81**	0.66, 0.97
Vancouver	1.03	0.91, 1.15	1.16**	1.01, 1.31	0.75***	0.64, 0.86
Victoria	3.17***	2.06, 4.86	3.35***	2.19, 5.11	0.91	0.67, 1.22
						
Observations	51,540		38,943		32,949	
Population size	11,016,136		8,491,902		6,930,585	
Pseudo R2	0.0312		0.0301		0.0363	
Wald chi2	1070.45	(df = 40)	833.49	(df = 40)	613.40	(df = 40)

^a ^Notes: The dependent variable is walking to work, school or errands. The model used for estimation is logistic regression. Bootstrap confidence intervals are listed. (*) significant at 10%, (**) significant at 5%, (***) significant at 1%. Reference categories are included in the table.

In Model 2, (lower-duration walking/no walking) the results of the logistic regression are generally consistent with those in Model 1. People with diabetes (OR = 1.30, 95% CI: 1.06, 1.59), the physically active (OR = 1.16, 95% CI: 1.09, 1.23), females (OR = 1.45, 95% CI: 1.36, 1.54), those aged 20 to 34 (OR = 1.25, 95% CI: 1.15, 1.35) and people in the two lowest income categories (OR = 1.39, 95% CI: 1.20, 1.59 and OR = 1.17, 95% CI: 1.07, 1.27) were all more likely to be lower-duration walkers. This type of walking was again found to be higher in the CMAs listed above (Toronto, Halifax, Hamilton, Regina, Calgary and Victoria) as well as in several additional urban areas, namely St. John's (OR = 1.46, 95% CI: 1.03, 2.06), Montreal (OR = 1.48, 95% CI: 1.32, 1.66) and Vancouver (OR = 1.16, 95% CI: 1.01, 1.31).

A different set of trends is apparent in Model 3 when walking duration (high/lower) is examined. In 2001, people with diabetes were less likely to be high-duration walkers (6 or more per week) than those who do not have this disease (OR = 0.79, 95% CI: 0.62, 0.99). Adults with a normal BMI were more likely to be high duration walkers than people who were overweight/obese (OR = 1.07, 95% CI: 1.00, 1.15) and the physically active were more likely to be this type of walker than the physically inactive (OR = 1.09, 95% CI: 1.01, 1.16). It should be noted that the odds ratios for three of the four socio-economic variables in Model 3 (sex, age, education) reverse their direction from Models 1 and 2. This outcome can be expected as the reference group in Model 3 (lower-duration walkers) changed from Models 1 and 2 (non-walkers). Females were less likely than males to be high-duration walkers (OR = 0.89, 95% CI: 0.82, 0.95) and adults aged 20 to 34 were less likely than those aged 50 to 64 to be high-duration walkers (OR = 0.90, 95% CI: 0.82, 0.99). Unlike Models 1 and 2, people with lower levels of education (less than high school, OR = 1.20, 95% CI: 1.03, 1.40; high school, OR = 1.40, 95% CI: 1.25, 1.55 and college and trades, OR = 1.38, 95% CI: 1.27, 1.49) were more likely to be high-duration walkers when getting to work. Similar to the first two models, people residing in households with lower incomes (under $20,000, OR = 1.44, 95% CI: 1.24, 1.65 and $20,000 to $49,999, OR = 1.23, 95% CI: 1.11, 1.35) were more likely to be high duration walkers. From an urban-regional perspective, Model 3 reveals that just three CMAs had significant odds ratios above 1: Winnipeg (OR = 1.35, 95% CI: 1.07, 1.69), Regina (OR = 1.33, 95% CI: 0.99, 1.78) and Calgary (OR = 2.09, 95% CI: 1.68, 2.60).

Table 4 displays the results of the 2005 logistic regression analyses and similar trends are apparent. However, unlike 2001, the presence of diabetes was not associated with walking in any of the three models. Consistent with the first study year, in 2005, adults with a normal BMI were 1.1 times more likely to be high-duration walkers (Model 3) when getting to work (OR = 1.11, 95% CI: 1.02, 1.19). In addition, physically active people were more likely to be walkers (Model 1, OR = 1.20, 95% CI: 1.13, 1.27) and lower-duration walkers (Model 2, OR = 1.18, 95% CI: 1.10, 1.26) than the physically inactive. In 2005, the socio-economic indicators associated with walking to work largely mirrored those in 2001. Again, females were more likely to be overall (Model 1) and lower-duration (Model 2) walkers but less likely to be high-duration (Model 3) walkers than males. The trends with respect to age, household income and education also remained consistent. From an urban-regional perspective, in 2005, Toronto along with several small to medium sized CMAs (Halifax, Kingston, Hamilton, Regina, Calgary and Victoria) were again prominent in having residents more likely to walk (Model 1) and be lower-duration walkers (Model 2) than people residing in non-CMAs. However, Table 4 highlights some changes with respect to this dimension. Winnipeg and Saskatoon were added to the list of CMAs having residents more likely to walk (Model 1) and to be lower-duration (Model 2) walkers. Also notable is the relative increase in the likelihood of walking to work in Vancouver in 2005 and the impressive growth (in terms of odds ratios) of walking to work in Victoria across all three models (Model 1, OR = 5.69, 95% CI: 3.81, 8.51, Model 2, OR = 5.10, 95% CI: 3.36, 7.73 Model 3, OR = 1.33, 95% CI: 1.03, 1.72).

**Table 4:** Results of Logistic Regression Analyses: Walking to Work, Adults aged 20 to 64 and Currently Working, 2005 ^a^

	Model 1 Walking/No Walking (all walkers = 1 non-walkers = 0)		Model 2 Lower-Duration Walking/No Walking (0 to 5 hrs/wk = 1 non-walkers= 0)		Model 3 High-Duration Walking/Lower-Duration Walking (6 or more hrs/wk = 1 0 to 5 hrs/wk = 0)	
Independent Variables	Odds ratios	95% CI	Odds ratios	95% CI	Odds ratios	95% CI
**Has diabetes**						
Yes	0.97	0.81, 1.15	0.95	0.79, 1.14	1.04	0.84, 1.29
No	Reference		Reference		Reference	
**Has high blood pressure**						
Yes	0.96	0.86, 1.06	0.95	0.84, 1.06	1.04	0.92, 1.17
No	Reference		Reference		Reference	
**Self-perceived stress**						
Not at all/not very	Reference		Reference		Reference	
A bit	1.11**	1.03, 1.18	1.11**	1.03, 1.20	0.98	0.90, 1.06
Quite a bit/extremely	0.97	0.89, 1.04	0.94	0.86, 1.02	1.08*	0.98, 1.18
**Adult BMI**						
Normal weight	1.01	0.94, 1.06	0.97	0.90, 1.03	1.11**	1.02, 1.19
Overweight/obese	Reference		Reference		Reference	
**Physical activity index**						
Active/moderate	1.20***	1.13, 1.27	1.18***	1.10, 1.26	1.03	0.96, 1.10
Inactive	Reference		Reference		Reference	
**Sex**						
Male	Reference		Reference		Reference	
Female	1.33***	1.25, 1.41	1.42***	1.32, 1.52	0.85***	0.79, 0.91
**Age**						
20-34	1.18***	1.09, 1.28	1.17**	1.06, 1.27	1.07	0.97, 1.18
35-49	1.02	0.94, 1.09	1.02	0.94, 1.11	1.01	0.92, 1.10
50-64	Reference		Reference		Reference	
**Household income**						
Under $20,000	1.63***	1.38, 1.91	1.48***	1.25, 1.75	1.30***	1.13, 1.49
$20,000 to $49,999	1.19***	1.10, 1.29	1.11**	1.01, 1.21	1.28***	1.16, 1.40
$50,000 to $79,999	1.08*	0.99, 1.16	1.04	0.95, 1.12	1.12**	1.02, 1.21
$80,000 and over	Reference		Reference		Reference	
**Education**						
Less than high school	0.71***	0.61, 0.83	0.61***	0.51, 0.72	1.52***	1.27, 1.81
High School	0.90**	0.81, 0.99	0.77***	0.68, 0.85	1.55***	1.37, 1.75
College or trades	0.89**	0.83, 0.96	0.81***	0.75, 0.86	1.35***	1.24, 1.45
University	Reference		Reference		Reference	
**Census Metropolitan Area**						
No CMA assigned	Reference		Reference		Reference	
St. John's	1.28	0.93, 1.74	1.29	0.91, 1.82	0.96	0.68, 1.35
Halifax	1.37**	1.03, 1.80	1.24	0.93, 1.65	1.29*	0.97, 1.69
Québec	0.59***	0.49, 0.71	0.74**	0.61, 0.89	0.46***	0.35, 0.60
Montréal	1.03	0.91, 1.16	1.27***	1.12, 1.43	0.53***	0.46, 0.60
Ottawa-Gatineau	0.97	0.82, 1.13	1.14	0.97, 1.33	0.59***	0.49, 0.72
Kingston	1.79**	1.27, 2.53	1.76**	1.22, 2.56	1.00	0.73, 1.37
Toronto	1.63***	1.45, 1.84	1.77***	1.56, 2.00	0.82**	0.72, 0.92
Hamilton	1.97***	1.59, 2.42	1.98***	1.58, 2.48	0.97	0.78, 1.20
London	1.32**	1.06, 1.63	1.28**	1.02, 1.61	1.08	0.83, 1.39
Thunder Bay	0.53***	0.39, 0.70	0.56***	0.40, 0.78	0.87	0.53, 1.40
Winnipeg	1.46***	1.18, 1.79	1.56***	1.24, 1.96	0.84	0.66, 1.06
Regina	2.84***	1.70, 4.73	2.77***	1.63, 4.69	1.07	0.83, 1.38
Saskatoon	1.53**	1.10, 2.13	1.36*	0.95, 1.93	1.32**	0.99, 1.76
Calgary	1.35**	1.09, 1.66	1.34**	1.09, 1.66	1.02	0.80, 1.28
Edmonton	1.06	0.86, 1.30	0.99	0.79, 1.22	1.16	0.91, 1.46
Vancouver	1.29***	1.11, 1.49	1.46***	1.25, 1.69	0.71***	0.61, 0.83
Victoria	5.69***	3.81, 8.51	5.10***	3.36, 7.73	1.33**	1.03, 1.72
						
Observations	50,082		36,903		34,865	
Population size	11,404,185		8,597,009		8,001,087	
Pseudo R2	0.0256		0.0279		0.0223	
Wald chi2	693.62	(df = 42)	620.41	(df = 42)	452.89	(df = 40)

^a ^Notes: The dependent variable is walking to work, school or errands. The model used for estimation is logistic regression. Bootstrap confidence intervals are listed. (*) significant at 10%, (**) significant at 5%, (***) significant at 1%. Reference categories are included in the table.

## Discussion

This paper examined the health, socio-economic and urban-regional characteristics of walking to work among adults in Canada in 2001 and 2005. The characteristics associated with walking were generally consistent over the study period indicating that from a cross-sectional perspective, the main findings are robust and can be generalized over time. However, there were several notable exceptions. From a health perspective, in 2005, there were no differences among the three study groups (non-walkers, lower-duration, high-duration) with respect to the presence of diabetes or high blood pressure. The primary health benefit, apparent in both years, was that adults within the normal weight range were more likely to be high-duration walkers. Not surprisingly, physically active people were also more likely to be walkers. Consistent with the findings of previous research, women, younger people and those with lower-incomes were more likely to be walkers or lower-duration walkers. However, these characteristics shifted when examining high-duration walkers with men, older adults, and those with lower-levels of education (along with lower-incomes) more likely to spend 6 or more hours a week walking to work.

The research demonstrated that there is a strong association between SES and walking. It has been well documented that people with lower incomes and less education are more likely to have health problems such high blood pressure and diabetes [22,23]. One can speculate that for many people, walking is a necessity rather than a choice and it is possible that the negative health factors associated with SES negate the health benefits of walking. In other words, a person who walks extensively during the average work week may not receive the full health benefits of this activity because of factors related to his or her relative social disadvantage such as poorer diet, less healthy lifestyle choices, work stress, or lack of access to recreational opportunities. This situation presents a challenge for policy makers and health practitioners; promoting walking (and other activities) as an appropriate form of exercise while managing the negative health outcomes associated with lower SES.

From an urban-regional viewpoint, the regression modeling found that walking was more prevalent in Canada's largest city, Toronto, and in several small to medium sized CMAs. Of these, Halifax, Kingston and Victoria, are characterized by a compact urban form, higher inner-city density and less suburban sprawl, factors which encourage walking. In the case of Victoria, a warmer climate and a network of recreational pathways facilitate this activity. A challenge remains in the promotion of walking and walking to work in cities and regions where rates are lower. Part of the solution lies in municipal planning, design and engineering as related to urban development and transportation infrastructure. Recent developments in Victoria can serve as a guide to other cities. In July 2008, the City's Engineering Department produced a Pedestrian Master Plan that was endorsed by City Council in October 2008 [24]. The plan is currently being implemented and will make sidewalks, pathways and crosswalks safer and more accessible and will improve lighting. It will also prioritize the construction of new sidewalks and establishes guidelines for sidewalk widths, streetscaping, accessibility and maintenance, including the prompt clearing of snow and ice in the winter.

Primary health care also has an important role to play in encouraging more walking. Family doctors often recommend that patients engage in exercise to improve their overall health or to deal with certain chronic medical conditions. Provincial or regional health authorities can promote the health benefits of walking and walking to work by consulting with family doctors and other primary health care practitioners working in private practices, community health centers or walk-in clinics. Emphasis can also be placed on public education through media advertising and dissemination aimed directly at schools and workplaces. However, Jane Hart, a physician, cautions that one message that doctors should not give to their patients is that any amount of walking -- no matter how little -- is enough to prevent disease or improve health [25]. She explains that people with limitations in their ability to exercise or who have disabilities may walk simply to maintain flexibility or reduce stiffness. Hart also proposes that to truly make a difference in many specific health measures such as high blood pressure or diabetes, some of a person's physical activity must be at least moderately vigorous in both duration and intensity. A number of studies on walking support this assertion [26 27 28]. To this end, it is commonly recommended that a person engage in at least 2.5 hours of moderately vigorous walking per week [25].

There are several limitations to this research. One issue is that the CCHS question asks broadly about walking to work, school, or for errands. While this paper narrowed the sample to people who are employed, a more direct measure of the time and distance associated with walking to and from work, apart from other daily activities, would be useful in refining the research parameters. A second limitation is that the physical activity data in the CCHS were self-reported and not directly measured which can lead to the over-reporting or under-reporting of activity levels, including time spent walking. Also, the study was cross-sectional and, as a result, the relationships are not causal - to establish causal relationships between walking to work and socio-economic and health predictors a longitudinal study would be required. A third limitation is that key indicators influencing the decision to walk, such as the number of vehicles in a household, having a driver's license and access to public transit are not collected in the CCHS. Future research could deal with some of these issues through the design of a specialized survey that deals directly with walking to work and the daily walking routines of a targeted sample of respondents in a single city. Such research may also help to shed light on the complex association between SES, health and the amount of time adults spend walking to work in an average week. From a methodological perspective, further research could be directed at using data from upcoming releases of the CCHS (for example, 2008/09). The data analysis could involve a number of regression methods (including generalized ordered logit models) to examine various cut-points in walking duration and expanding the number of categories across the non-walking to walking continuum.

## Conclusions

As stated, from a non-medical perspective, many people are limited in their ability to walk to work due to distance or lack of safe routes. However, innovative strategies can be considered to encourage people to incorporate more walking into their daily routines. One of these is the concept of mass transit walking, which involves providing incentives for people to take public transit to work rather than drive, thereby automatically building walking into a person's daily commute. Research has shown that people who walk from their home to a transit stop and then to their place of work are more physically active and have significantly better health outcomes than those who drive directly [29,30]. The Atlanta Clean Air Campaign has developed a mass transit-walking program that has proven to be effective. Essentially, the program pays participants $3 a day to take mass transit as long as they record the daily mileage of their train trips using an on-line calculator [31].

For a large number of Canadians, public transit is not an option for their daily commute; it is either not available (as is the case in many rural and remote regions) or, due to lifestyle choices, there is an unwillingness to take it regardless of the incentives offered. In the 2006 Census, more than 10.3 million people (about 80% of commuters) said they took a vehicle to work, either as driver or passenger. For people in this group who are concerned about maintaining or reducing their weight and improving their health, a workplace-walking program may be an option. At the same time, it is in the interest of employers to encourage physical activity. Research has consistently shown that workers who are physically and psychologically healthy are more productive and will ultimately improve an organization's 'bottom line' [32]. A simple and cost-effective approach is for employers to set up a formal program where employees are encouraged to walk during the day, especially at lunch and during breaks. Walking programs have been set up in a number of workplaces and have proven to be effective. Typically, employees enroll, are issued a pedometer and given a time frame (usually in months) in which to gradually increase the number of steps they take in a day, with the ultimate objective of reaching 10,000, a level that is considered to be an active lifestyle [25]. These programs are normally run in a fun and supportive manner. Walk BC is an initiative based in the Canadian province of British Columbia, which promotes workplace walking. Walk BC is a joint effort between the Heart & Stroke Foundation of BC & Yukon and the BC Recreation & Parks Association (http://www.walkbc.ca). It has designed a detailed 12-month workplace-walking program, which includes monthly walking themes (e.g. walk at lunch, climb stairs, walk in the park) and monthly goals (increasing duration and pace).

## Competing interests

The authors declare that they have no competing interests.

## Authors' contributions

PK designed and conceptualized the study and conducted the data analysis. AW conducted the literature review and contributed to the discussion and conclusion sections. JC performed the logistic regression models in Stata 11. All authors have read and approved the final manuscript.

## Appendix 1: Footnotes

^1 ^The Physical Activity Index is a derived variable. The CCHS categorizes respondents as being 'active', 'moderate', or 'inactive' based on total daily energy expenditure values in kcal/kg/day (KKD): Inactive (KKD less than 1.5); Moderate (KKD 1.5 to 2.99); Active (KKD of 3 or greater).

Energy Expenditure is calculated using the frequency and duration per session of the physical activity as well as the MET value of the activity. The MET is a value of metabolic energy cost expressed as a multiple of the resting metabolic rate.

EE (Energy Expenditure for each activity) = (N X D X METvalue)/365

Where:

N = the number of times a respondent engaged in an activity over a 12 month period

D = the average duration in hours of the activity

MET value = the energy cost of the activity expressed as kilocalories expended per kilogram of body weight per hour of activity (kcal/kg per hour)/365 (to convert yearly data into daily data)

## Pre-publication history

The pre-publication history for this paper can be accessed here:

http://www.biomedcentral.com/1471-2458/11/212/prepub
